# Transient Increase and Delay of Multifocal Electroretinograms Following Laser Photocoagulations for Diabetic Macular Edema

**DOI:** 10.3390/jcm10020357

**Published:** 2021-01-19

**Authors:** Yoshiaki Shimada, Masayuki Shibuya, Kei Shinoda

**Affiliations:** 1 Department of Ophthalmology, Fujita Health University, 1-98 Dengakugakubo, Kutsukake-cho, Toyoake, Aichi 470-1192, Japan; 2 Department of Ophthalmology, Saitama Medical University, 38 Morohongo, Moroyama-machi, Iruma-gun, Saitama 350-0495, Japan; arainko5@yahoo.co.jp (M.S.); shinok@saitama-med.ac.jp (K.S.)

**Keywords:** diabetic macular edema, diabetic retinopathy, electroretinogram (ERG), multifocal electroretinogram (mfERG), photocoagulation

## Abstract

Background: The acute physiological changes induced by focal retinal photocoagulation (PC) have been largely unexplored. Methods: This was a case-series study. We recorded multifocal electroretinograms (mfERGs) just before PC, and mfERGs were also recorded 5′, 15′, one hour, 24 h, and one week after the PCs. Transient changes of mfERGs were analyzed in eyes which underwent PCs to treat diabetic macular edema. The mfERGs recorded from the predominantly irradiated area and that from non-irradiated areas were analyzed separately. Results: Fifteen eyes of 15 patients were included in this study. The mfERGs elicited from non-irradiated areas did not change after PC, but the mfERGs elicited from the irradiated area changed with time; the amplitude was larger at 60′ than that before (*p* < 0.05) and at 5′ after PC (*p* < 0.01) and significantly smaller at 24 h and 1 week than that before and at 60′ after the PC (*p* < 0.01). The implicit time was significantly prolonged after PC. mfERG on irradiated area with the severe diabetic change was less altered after PCs. Conclusions: The transient increase in the amplitude at 60′ likely resulted from a biological amplification of partially damaged cells adjacent to the PC spots. The mfERGs manifested the dynamic alterations of the retinal function following PCs.

## 1. Introduction

Retinal photocoagulation (PC) has been used extensively to treat various retinal disorders, and its effectiveness in preserving vision has been established [1,2]. PC is performed typically with laser light, which raises the temperature in the irradiated areas and leads to necrotic cell death. Although the exact mechanism for this is still largely unknown, the beneficial effects of PC are believed to be related to the retinal damage caused by PC, especially due to improvement in inner retinal oxygenation [1,2].

For decades, electroretinograms (ERGs) have been used to assess the alterations of retinal function caused by PC [3,4,5,6,7,8,9,10,11,12,13,14,15,16,17,18,19,20,21,22,23,24,25,26,27,28,29,30,31] to treat diabetic retinopathy (DR) or diabetic macular edema (DME) [2,3,4,5,6,7,13,14,15,16,19,21,23,24,25,27,29,30,31], central serous chorioretinopathy (CSC) [20,22] and animal models [8,10,11,12,17,18,26,28].

However, most of these ERG studies recorded ERGs after several to 24 h after PC, which is long after the acute changes had occurred [4,9,11,12,28,31]. ERGs have also been recorded at even longer intervals after PC [3,5,6,7,8,9,10,13,14,15,16,17,18,19,20,21,22,23,24,25,26,27,28,29,31] to assess the therapeutic effects of PCs.

On the other hand, acute ERG changes immediately after PC may provide a novel way to differentiate PC effectiveness variations. Focal ERG [32] and multifocal ERGs (mfERGs) [33] recorded during and immediately after transpupillary thermotherapy (TTT), respectively, could depict the very acute functional changes during and immediately after laser irradiation. In this research, we analyzed mfERGs sequentially obtained right after the PC to explore acute functional changes in the targeted and the non-targeted surrounding retina induced by the PC.

## 2. Experimental Section

### 2.1. Methods and Patients

The study protocol and procedures conformed to the tenets of the Declaration of Helsinki and were approved by the Institutional Review Board of Saitama Medical University Hospital (SMUH, IRB No. 20133.01). We investigated clinical records and mfERGs. Between January 2006 and January 2007 at SMUH, patients who underwent focal PC to treat a macular disease for the first time were asked to undergo recording mfERGs before and after PC. Eyes with significant cataract or vitreous opacity/hemorrhage and eyes which had previously been treated with PC were excluded.

Among thirty-seven patients who provided written informed consent to and completed the protocol, fifteen eyes of 15 patients (7 men and 8 women, 59.7 ± 8.6 years old, mean ± standard deviation) were for DME (Table 1). The remaining 22 patients were not for DME, most were for CSC.

This chart review was done prior to anti-VEGF therapy availability, thus anti-vascular endothelial growth factor (VEGF) therapy was not used, and the PC was the first line therapy for all patients. The identification of DME was made by ophthalmoscopy, fluorescein angiography (FF450plus, Zeiss Humphrey Systems, Dublin, Ireland), and optical coherence tomography (OCT; OCT 3000; Zeiss Humphrey Systems). Adult patients with type 1 or type 2 diabetes mellitus who presented with central-involved DME (defined as retinal thickening involving the 1 mm central subfield thickness) were included.

The retinal areas that would be treated by PC had microaneurysms with exudates that were detected by fluorescein angiography. OCT images showed retinal thickening at the site of the lesions, but a serous retinal detachment was not detected.

The Novus^®^ Omni™ laser (Lumenis, Santa Clara, CA, USA) was used for the PC. The parameters for PC were as follows: 14 to 31 shots at 647 nm wavelength, 0.15–0.2 mm diameter, 0.15–0.2 W power, and 0.2′′. duration. PC was performed with a Goldmann 3-mirror lens, and the procedures were completed in less than 3′. The coagulated spots appeared as faint grayish-white spots barely visible by ophthalmoscopy.

### 2.2. Multifocal Electroretinograms (mfERGs)

mfERGs were recorded with the VERIS™ Science 5.1.12 system (EDI: Electro-Diagnostic Imaging, San Mateo, CA, USA). The recordings were performed under ordinary room light with a maximally dilated pupil. The stimuli were displayed on a monochrome CRT monitor with a P4 white phosphor. The stimuli consisted of an array of 37 contiguous hexagons. The overall stimulus subtended 40° of the central visual field (hexagons were scaled with a stretch factor, 13.18). An m-sequence rate of 75 frames/s and a cycle of 2^14^-1 steps resulted in a net recording time of 3′38′′. A camera/refractor™ (EDI) was used to refract the subject and to monitor the eye position and fixation during the recordings [33,34].

Signals were picked up by a GoldLens™ (Diagnosys LLC, Littleton, MA, USA), amplified (100,000 ×), bandpass filtered (10–300 Hz at half-amplitude), and digitalized at a 1200 Hz sampling frequency.

After recording the mfERGs, the GoldLens™ was replaced by the Goldmann lens to perform the PC. At the completion of the PC, the lenses were interchanged and mfERGs were recorded at different times after the PC. Each recording took approximately 4’. The earliest recording was started at one minute and was completed at 5′ after the PC. The second and third recordings were at 11–15′ and 55–60′, respectively. The times of these recordings are designated as 5′, 15′, and 60’, respectively. mfERGs were also recorded on the following day (24 h) and at one week (1 week), for a total of 6 sets of mfERGs.

**Figure 1:**
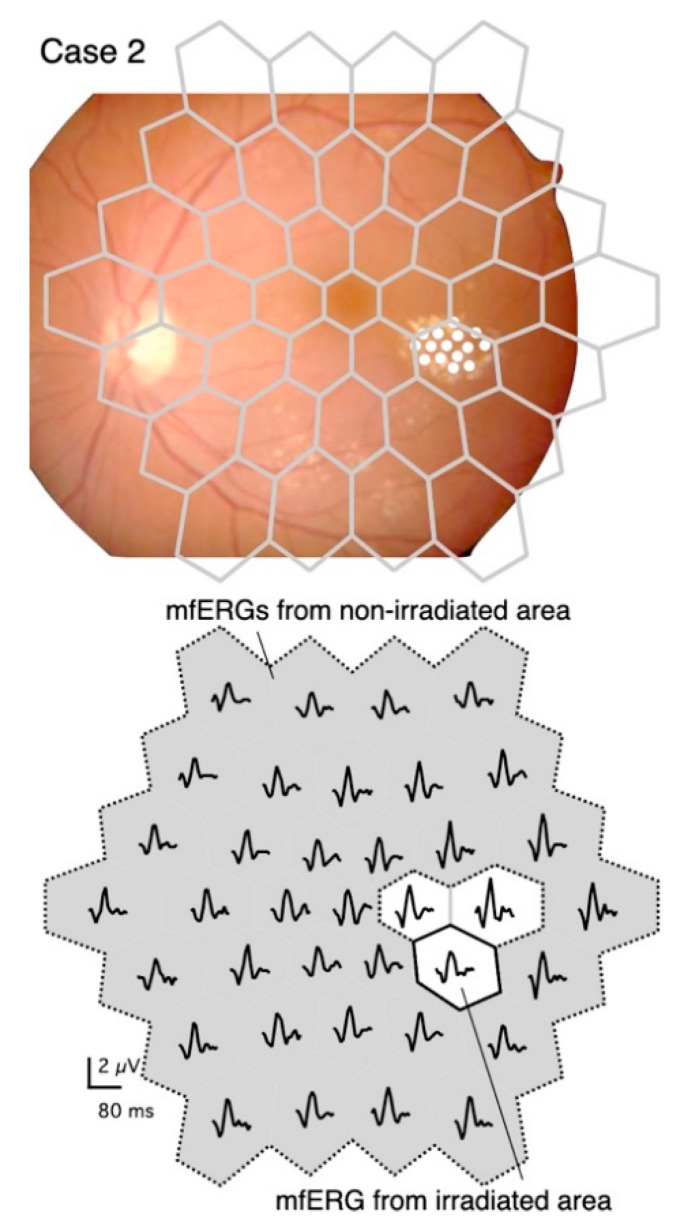
Stimulus pattern superimposed on a fundus image, and distribution of multifocal electroretinograms (mfERGs) recorded at different retinal sites.

The upper panel in Figure 1 shows that the stimulus pattern is superimposed on a fundus image of the left eye of a 56-year-old woman (Case 2) in order to demonstrate the location of the stimuli on the retina. The fundus image was flipped vertically so that the mfERGs corresponded to the stimulus pattern (Figure 1, lower panel).

Upper panel: Diagram of stimulus consisting of 37 stimulus hexagons superimposed on the fundus of Case 2. The fundus image is flipped vertically to correspond to the mfERG traces shown in the lower panel. The diabetic changes were mild except for the temporal perifoveal exudates, which were targeted for PC. The coagulated spots were barely visible in the fundus image, so they are marked with white circles.

Lower panel: The irradiated area (solid bordered) and the non-irradiated area (gray with dashed border). The boundary regions (white without solid border) were excluded from the analyses.

The mfERGs elicited from the stimulus elements that fell on the predominantly irradiated retinal region are designated as “mfERGs from the irradiated area” (Figure 1, lower panel). The mfERGs obtained from stimulus elements that were completely separated from the irradiated area were summed and designated as “mfERGs from a non-irradiated area”. Responses from the boundary regions that might overlap the coagulation spots were excluded from the analyses.

The amplitude of the mfERGs, i.e., the voltage between the first negative and the first positive peak expressed as the response density (nV/deg^2^), and the implicit time (ms) of the first positive peak were used for the analyses. The amplitude and the implicit time of the response from “mfERGs from the irradiated area” and “mfERGs from the non-irradiated area” were calculated and used for statistical analysis. Transitions, 2-combination from 6 time points; 15 comparisons of the amplitude and the implicit time were statistically analyzed by a paired *t*-test with Bonferroni correction to reduce the type I error introduced by multiple comparisons. A *p*-value less than 0.05 was considered statistically significant.

The average mfERGs were recorded from eight, age-matched (58.3 ± 10.7 years old), healthy subjects. The normative mfERGs included five men (44, 51, 56, 58, and 65 years), and three women (48, 70, and 74 years), whose visual acuities were ≥20/20. The normative mfERGs had not been included in the statistical analyses.

## 3. Results

**Figure 2:**
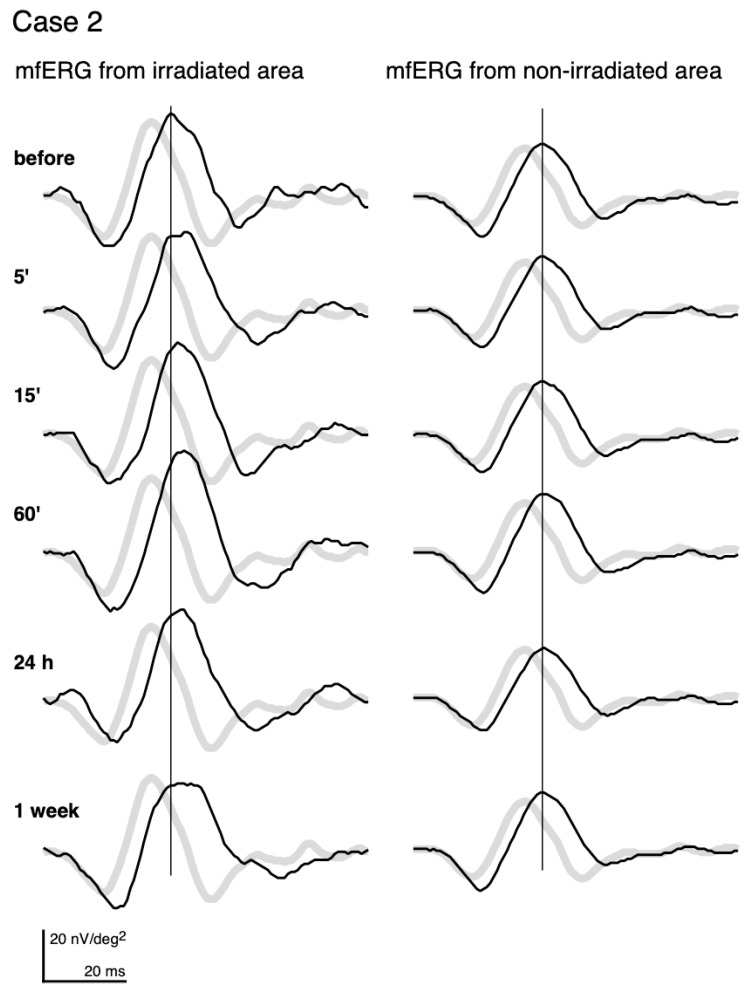
mfERGs recorded before and at different times after photocoagulation (PC). mfERGs from the irradiated area (**left**) and from the non-irradiated area (**right**) of Case 2 (same case as Figure 1) are shown. The pale curves represent the averaged normative mfERGs obtained from 8 healthy volunteers at the same loci.

The mfERGs recorded from the irradiated area (left column) and those from a non-irradiated area (right column) of the same eye of Case 2 are shown in Figure 2. The pale waveforms represent the average responses in the corresponding locations of the irradiated and non-irradiated areas of normative mfERGs.

Even before PC, the mfERGs from both the irradiated area and non-irradiated area had longer implicit times relative to the normative mfERGs. We assume that this is due to diabetes or DR. This pre-irradiation delay was commonly observed. The delay was more than 0.83 ms of the implicit time of the first positive peak in all subjects for the mfERG recorded from the irradiated area. In addition, the delay was found in the mfERGs recorded from the non-irradiated areas in 13 of the 15 subjects.

After PC, the mfERGs from the non-irradiated areas (right column) did not show any changes throughout the recordings. Statistical analyses of the average and each individual patient did not have any significant alterations of the amplitudes and implicit times in the non-irradiated areas.

On the other hand, the mfERGs from the irradiated area (left column) altered after PC. The implicit time was more prolonged at 5’, and the delay became more distinct and did not recover to the pre-PC level until 1 week later. The amplitude of the mfERGs from the irradiated area got larger at 15’ and 60’, and then decreased at 1 week.

**Figure 3:**
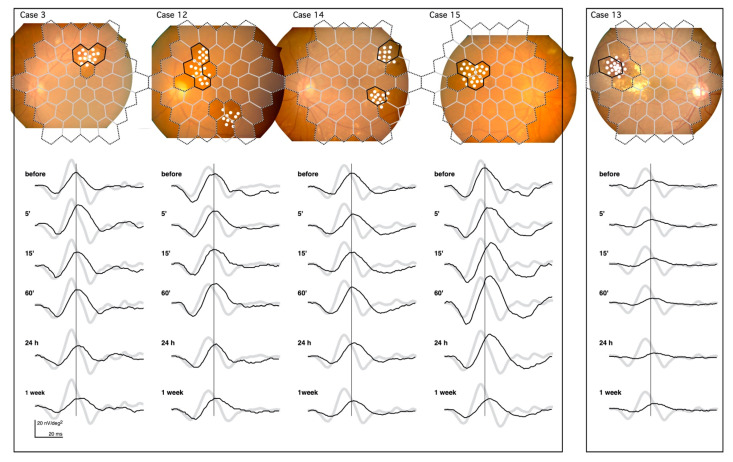
mfERGs recorded from the irradiated area of five cases. The solid borders superimposed on the fundus images represent the location of the mfERGs from the irradiated areas. Dashed borders represent the non-irradiated areas. The mfERGs were unchanged throughout the recordings (not shown). The boundary regions without solid borders were excluded from the analyses. The pale mfERGs represent the mean normative mfERGs obtained from 8 healthy volunteers. Note that the PC was performed at different retinal sites in each case, and the normative mfERGs were obtained from the corresponding retinal sites.

The mfERGs recorded from Cases 3, 12, 14, and 15 are shown in the left panel of Figure 3. They demonstrate the same pattern of changes after the PC as Case 2 in Figure 2.

Left panel: Cases 3, 12, 14, and 15 demonstrate the typical mfERG changes after PC. This shows the prolongation of the implicit time and increase in the amplitude at 60’, followed by the late attenuation.

Right panel: Case 13 had the most severe exudative changes in the irradiated area, and had the least amount of change in the mfERG after PC.

The prolongation of the implicit time and the increase in the amplitude followed by the late attenuation can be seen in these cases.

The changes in the mfERGs after the PC in Case 13 (right panel in Figure 3) were the smallest changes recorded of all subjects. The circinate exudates in this case were centered on the microaneurysms and were surrounded by subretinal hemorrhage that almost filled the entire irradiated area. The retina in the irradiated area was damaged even before PC. This is seen in the reduced mfERG amplitude, and the damage present is likely permanent, as there were no changes following PC. The pathology underlying the DME varied in this study; however, we did not see a lesion more severe than that of Case 13.

**Figure 4:**
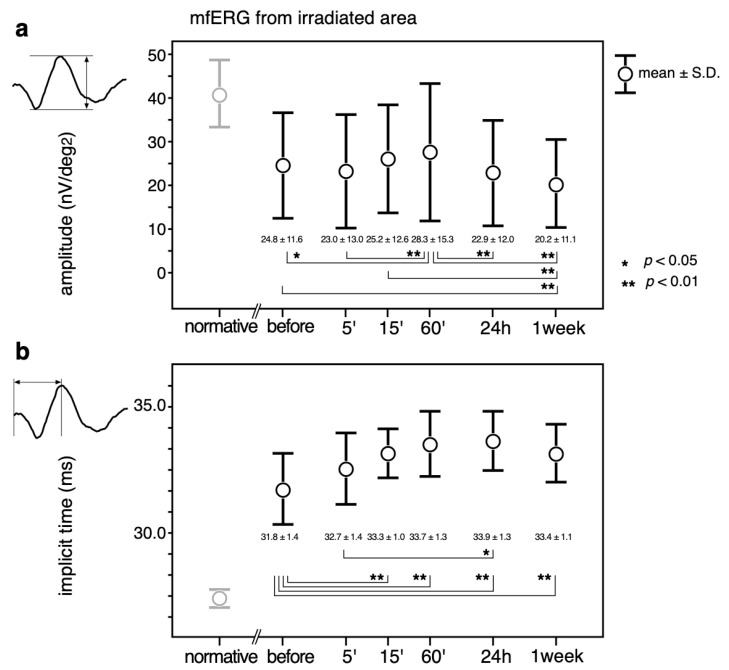
Mean ± standard deviations over the time course of the changes in the amplitudes (**a**) and implicit times (**b**) of the mfERGs recorded from the irradiated area. Transitions, 2-combination from 6 time points; 15 comparisons were statistically analyzed by a paired *t*-test with Bonferroni correction. A *p* value less than 0.05 (*) was considered statistically significant. A *p*-value less than 0.01 was marked with **. Normative mfERG obtained from 8 healthy volunteers are also plotted (left most column, pale circles and error bars) to show that the mfERG from the irradiated area was altered by diabetic changes even before the PC. Normative values were not included in the statistical analysis.

The mean ± standard deviations over the time course of the changes in the amplitudes are shown in the upper panel (a) in Figure 4. The implicit times (Figure 4, b) of the mfERGs recorded from the irradiated area are also shown.

The largest amplitudes were recorded at 60’, which was significantly larger than those before (*p* < 0.05) and at 5’, 24 h, and 1 week (*p* < 0.01) after PC. The amplitude at 1 week was significantly smaller than the amplitude before and at 15’ and 60’ after the PC (*p* < 0.01).

The implicit times became even longer after the PC, and the differences were statistically significant at all testing times except at 5’ after PC (*p* < 0.01). The implicit time was significantly longer at 24 h than at 5’ (*p* < 0.05).

## 4. Discussion

### 4.1. Rationale for Recording mfERGs Immediately after PCs

We had hypothesized that recording the mfERGs immediately after the PC would reveal the early physiological changes in the retina. Earlier studies using full-field ERGs [3,4,5,6,7,8,9,10,11,12,13,14,15,16,17,19,24,29,30,31] could not determine the functional alterations produced by a small PC spot. Therefore, investigators typically examined subjects who had hundreds of coagulation spots as in panretinal PC. This procedure required tens of minutes to complete, so the temporal resolution was lower. Additionally, the artifacts of the PC procedures, such as long periods of elevated intraocular pressure from pressure of the contact lens, and the effects of light adaptation due to the laser irradiation, confounded the observations.

The use of mfERGs [18,20,21,22,23,25,26,27,28] enables assessment of the retinal region predominantly coagulated separate from the region not coagulated. In addition, unlike the conventional ERGs, which depend on the state of adaptation, mfERGs with a high base rate, such as 75 Hz, are known to induce a very high level of light adaptation quickly during the recordings [34,35]. Thus, the differences in the state of adaptation before and after PC can be minimized.

Importantly, the mfERGs from the non-irradiated areas of all eyes did not change after PC as had been observed with mfERGs following TTT [31]. This shows that the alterations in the mfERGs recorded from the irradiated area are valid physiological changes induced by the PC.

### 4.2. Transiently Large and Delayed mfERGs after PC

The amplitude at 5’ after PC tended to be smaller than that before PC. However, at 60′, the amplitude was significantly larger than before, and at 5’ after, the PC. Following TTT, the amplitudes of the focal flicker ERGs [32] and mfERGs [33] during [32], and 1’ after [33] the irradiation, were significantly smaller due to the direct thermal effect on the retina. This reduction disappeared quickly [32,33]. Our earliest post-PC mfERG recording could be completed at 5′, and this may explain why we did not find the very early attenuation in the present data.

The amplitude was larger at 60’ with a significant prolongation of the implicit time. While the coagulated areas had a higher reflectance, which may enhance the stray light effects [34], our coagulation spots were barely visible. Thus, we can safely assume that the stray light effects were minor. A transient increase of mfERGs after PC has been reported by Ver Hoeve et al. [IOVS 2004; 45: ARVO E-Abstract 4239]. They recorded mfERGs of monkeys at 60’ after macular grid PC and found large mfERGs with prolonged implicit times followed by a reduction of the response. Their coagulation spots were whitish; however, they showed that the amplitude of the second-order kernels (K2.1) also increased. This suggests that the enhancement was not due to stray light effects [34]. Our results indicate that the large and delayed mfERGs after PC can also be seen in human subjects.

Our subjects had DR/DME, thus mfERGs were diabetically altered. Compared to the normative mfERGs, the implicit time was longer and the amplitude was smaller in the mfERGs recorded from both the irradiated and non-irradiated areas before PC. The prolongation of the implicit time, and the attenuation of the amplitude, are reported to reflect functional impairments of the retina caused by DR [36,37,38]. We assume that the transient amplitude increase would be much more apparent if the diabetic impairments did not exist, because the increase was not observed in eyes whose mfERGs were severely attenuated due to diabetic change, such as that in Case 13.

We wondered how mfERGs can be larger and most apparently delayed 60’ after the PC. We hypothesize that the photoreceptors in a coagulation spot were killed within seconds by PC [39], and thus, the mfERG changes after PC originated from the cells in the adjacent regions. We suspect that this is probably because of a biological amplification in partially damaged structures that can still react [39]. Heat transfer or biochemical mediators from coagulation spots alter photoreaction in the adjacent regions that can emerge as large mfERG; for instance, alterations in retinal pigment epithelium (RPE) resistance can produce significant changes in b-wave amplitude [40].

Some mediators have reportedly increased after PC [41,42,43,44,45,46,47,48,49,50,51,52,53]. Prostaglandin E2 appeared in aqueous humor after iris PC in rabbits and it peaked 30–90’ after PC [41]. Prostaglandins and prostaglandin-like substance were assumed to mediate acute post-PC reactions [41,42,43,45,46].

Nitric oxide (NO) synthase inhibitor suppressed the protein increase in aqueous humor 30’ after iris PC in rabbits [44]. NO was thought to have contributed to post PC reactions [44,45,46,49], such as increased vasodilation of retinal vessels after retinal PC [47].

Heat shock protein (Hsp) 70 mRNA expression was induced within 30’ of laser irradiation, peaking at 180’ after laser irradiation in a single layer of densely cultured human ARPE-19 cells [51]. HSPs [47,48,49,50,51,52,53], especially Hsp70 [47,48,50,51,52,53], are suggested to be one of the key factors in the therapeutic effect of hyperthermia treatments. How each of these factors contributes to the acute mfERG alterations warrants further investigation.

### 4.3. Limitations, Clinical Application and Suggestions

There were limitations to the current study. First, inclusion criteria were patients who underwent focal PC to treat DME for the first time and completed the protocol, regardless of their age, gender, the general state of diabetes and DR, pre- and postoperative visual acuities and types of DME. Some information was no longer available, so that influence of those factors remains unknown. Moreover, the PC treatments were performed prior to anti-VEGF therapy availability; that has changed the paradigm in the treatment of DME [25,27,31]. Indication and procedures of the PC have been continuously changing.

ERG studies have classically investigated the role of spot size [4], irradiation duration and power [9], and wavelength [6,7]. More recently, they have investigated the difference in short-pulse and subthreshold PCs [23,28,29,30,31] and the influence of additional [25,31] or comparative [27] anti-VEGF agents to PC treatments. Acute mfERG changes immediately after PC may provide a novel way to differentiate PC effectiveness variations, especially in subthreshold PC, which is performed without making visible coagulation spots [23,29]. This may allow acute mfERG alterations to contribute to objective indexes post PC.

In conclusion, mfERGs can demonstrate early alterations in retinal function following PC for DME. The amplitude was transiently increased at 60’, then clearly attenuated at 1 week after PC. The implicit time was significantly prolonged after PC. mfERGs showed the dynamic alterations of retinal function following PC.

## Figures and Tables

**Table 1:** Summary of Subjects.

Case	Age/Gender	Eye	VA *	Photocoagulation †
1	65/F	OD	0.5	0.15 w, 0.20 s, 0.15 mm, 22 (11) shots
2	56/F	OS	0.8	0.15 w, 0.20 s, 0.15 mm, 15 (14) shots
3	67/F	OS	0.6	0.15 w, 0.20 s, 0.20 mm, 14 (13) shots
4	72/F	OS	0.7	0.20 w, 0.20 s, 0.20 mm, 20 (13) shots
5	78/F	OS	0.5	0.2 w, 0.2 s, 0.2 mm, 22 (11) shots
6	62/M	OD	1.0	0.15 w, 0.2 s, 0.15 mm, 18 (7) shots
7	61/M	OD	0.4	0.2 w, 0.2 s, 0.2 mm, 25 (19) shots
8	56/M	OS	1.0	0.2 w, 0.2 s, 0.2 mm, 20 (12) shots
9	61/F	OD	0.9	0.2 w, 0.2 s, 0.2 mm, 20 (16) shots
10	59/M	OD	0.6	0.2 w, 0.2 s, 0.2 mm, 29 (17) shots
11	57/M	OD	0.5	0.2 w, 0.2 s, 0.2 mm, 26 (19) shots
12	44/F	OS	0.7	0.2 w, 0.2 s, 0.2 mm, 31 (20) shots
13	54/M	OD	0.5	0.2 w, 0.2 s, 0.2 mm, 22 (18) shots
14	56/F	OS	0.7	0.2 w, 0.2 s, 0.2 mm, 23 (19) shots
15	48/M	OD	0.8	0.2 w, 0.2 s, 0.2 mm, 21 (19) shots

* best corrected visual acuity before the treatment; † laser out put, duration, spot size, number of shots; (number of spots within the area analyzed as irradiated area).

## Data Availability

The data presented in this study are available on request from the corresponding author.
